# Cluster of differentiation 30 expression in lacrimal gland and conjunctival tissues in patients with Sjögren's syndrome: Case series

**DOI:** 10.1097/MD.0000000000016390

**Published:** 2019-07-19

**Authors:** Akiko Ogawa, Yoko Ogawa, Shin Mukai, Eisuke Shimizu, Masataka Kuwana, Yutaka Kawakami, Kazuo Tsubota

**Affiliations:** a Department of Ophthalmology, Keio University School of Medicine, Tokyo, Japan; b Center for Interdisciplinary Cardiovascular Sciences, Brigham and Women's Hospital, Harvard Medical School, Massachusetts; c Department of Allergy and Rheumatology, Nippon Medical School; d Division of Cellular Signaling, Institute for Advanced Medical Research, Keio University School of Medicine, Tokyo; e International University of Health and Welfare School of Medicine, Chiba, Japan.

**Keywords:** cluster of differentiation 30, conjunctiva, lacrimal glands, malignant lymphoma, Sjögren's syndrome

## Abstract

**Introduction:**

Sjögren's syndrome (SS) often causes lymphoproliferative disorders such as malignant lymphoma and macroglobrinemia. Approximately 5% of long-term follow-up SS patients develop malignant lymphoma. Recently, the tumor necrosis factor receptor superfamily cluster of differentiation 30 (CD30) has been thought to be implicated in malignant cells in organs affected by Hodgikin lymphoma or in a prognostic marker of diffuse large B cell lymphoma. In this study, we investigated CD30 expression in lacrimal gland and conjunctiva in patients with SS.

**Methods:**

We examined lacrimal gland and conjunctival tissues for the diagnosis from 3 female SS patients with a median age of 51 and 3 female chronic graft-versus-host disease (cGVHD) patients with a median age of 41. Histological analysis of these tissues of the remaining samples was conducted by methods including immunohistochemistry and electron microscopy (#20090277). We analyzed the expression and localization of cluster of differentiation 4 (CD4), cluster of differentiation 8 (CD8), cluster of differentiation 20 (CD20), CD30, and Interferon-γ in tissue sections prepared from lacrimal glands and conjunctiva in 3 each of SS and cGVHD patients.

**Results:**

There were more B cells and plasma cells in lobules of SS-affected lacrimal glands than in those of their cGVHD-affected counterparts. Interferon-γ was expressed on endothelia of capillaries in SS-affected lacrimal gland and conjunctival tissues whereas it was expressed on fibroblasts in their GVHD-affected equivalents. Furthermore, lacrimal glands and conjunctiva disordered by SS had a greater number of CD30^+^ cells than those disordered by cGVHD.

**Conclusion:**

Our results suggest that CD30^+^ cells are increased in lacrimal glands and conjunctiva affected by SS and that a subset of SS patients are thereby at risk of development malignant lymphoma.

## 1 Introduction

Sjögren's syndrome (SS) is characterized by inflammatory cell infiltration into lacrimal glands, salivary glands, and other exocrine glands, leading to dry eye, dry mouth, and extra-glandular syndrome.^[1]^ SS is an autoimmune disease and a third of SS patients develop systemic complications including pulmonary, renal, neurological disorders, hematological, and musculoskeleton.^[2]^ There are 2 types of SS—one is primary SS without other connective tissue diseases, and the other is secondary SS accompanied by other connective tissue diseases such as systemic lupus erythematosus, rheumatoid arthritis, and/or systemic sclerosis.^[3]^

SS often causes lymphoproliferative disorders such as malignant lymphoma and macroglobrinemia. Approximately 5% of long-term follow-up SS patients develop malignant lymphoma.^[4]^ It has been reported that primary SS can be divided into 3 stages according to the extent of organ damage and the course of the disease. In stage I, (approximately 45% of SS cases), patients have only sicca syndrome without any other systemic involvement, even after 10 years. In stage II (approximately 50% of SS cases), patients have lymphocytic organ damage, which may involve the pulmonary, renal, hepatic, hematologic, and/or dermatologic systems, among others. In stage III (approximately 5% of SS cases), patients develop malignant lymphomas. Lymphomas in salivary glands are thought to arise from lymphoepithelial lesions in which there are close interactions between epithelial cells, T cells, and B cells. However, pathogenic line between lacrimal glands affected by SS and lypmphoma remains to be elucidated.

Cluster of differentiation (CD) 30, known as a member of the tumor necrosis factor receptor superfamily, is highly expressed on malignant cells in organs affected by Hodgikin lymphoma (HL) or anaplastic large cell lypmphoma (ALCL).^[5]^ Recently, CD30 has been thought to be implicated in an prognostic marker of diffuse large B-cell lymphoma (DLBCL).^[6,7]^

In this study, we investigated the augmentation in the expression of CD30 in SS-affected lacrimal gland and conjunctival tissues and discussed the significance of our findings in SS patients in clinical settings.

## 2 Methods

### 2.1 Patients and disease control subjects

The research ethical committee at Keio University approved this study (#20090277). Written informed consent was obtained from all patients in accordance with the principles expressed in the Declaration of Helsinki. Each patient has provided written informed consent for publication of the case. Lacrimal gland and conjunctival biopsies were applied to 3 patients with SS and 3 patients with chronic graft-versus-host disease (GVHD) for diagnostic purposes, and the remaining tissues were examined for analytical purposes in this observational case series.^[8]^ Clinical characteristics of these patients are shown in Table 1. Three female SS patients with a median age of 51 (range: 34–61) and 3 female GVHD patients with a median age of 41 (range: 24–47) were examined. At the time of biopsies, 5 patients (3 with SS and 2 with chronic GVHD) had severe dry eye. The diagnosis of SS was based on the Japanese Ministry of Health criteria revised in 1999.^[3]^ Severe dry eye was determined by the Schirmer test with nasal stimulation equal or <10 mm, fluorescein scores of 3 or more out of 9 and rose bengal scores of 3 or more out of 9. Histological analyses of these tissues were conducted by the methods including immunohistochemistry and electron microscopy. We analyzed the tissue structures using Hematoxylin & Eosin (HE) staining, and investigated the expression and localization of CD4, CD8, CD20, CD30, and Interferon-γ in tissue sections prepared from lacrimal glands and conjunctiva in 3 each of SS and GVHD patients (Table 2).

**Table 1:**
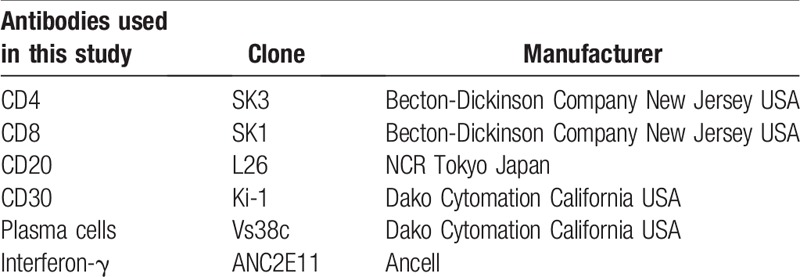
Antibodies used in this study.

**Table 2:**
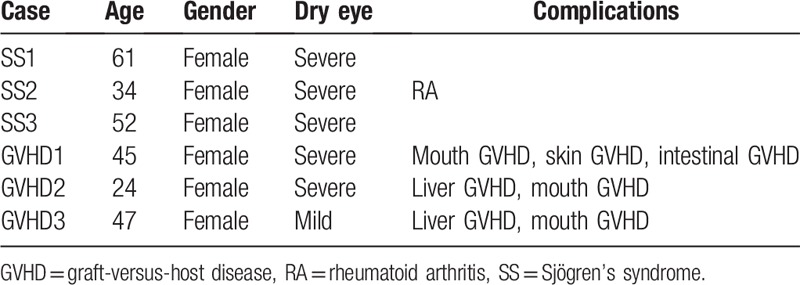
Clinical characteristics of patients enrolled in this study.

### 2.2 Immunohistochemistry

For paraffin sections, all lacrimal gland specimens were fixed in 10% neutralized buffered formalin immediately, embedded in paraffin wax, and processed according to histologic techniques, including HE staining. For immunohistochemistry, deparaffinized sections were incubated with 0.3% H_2_O_2_ for 5 minutes at room temperature to remove any endogenous peroxidase. The respective primary antibodies for B cells and plasma cells were added to the sections and incubated for overnight at 4 °C with a subsequent incubation of the corresponding peroxidase-conjugated secondary antibodies (En Vision; Dakopatts, Glostrup, Denmark). The bound antibody was visualized with diaminobenzidine tetrahydrochloride, and cell nuclei were counterstained with hematoxylin for 1 second. All incubation steps were performed in a moist chamber. Antigenic epitopes were unmasked using the antigen retrieval method described in Table 1. Briefly, for B cells and plasma cells staining, the slides were treated in a microwave oven for 10 minutes with a preheating process for 11 minutes.^[9]^

For frozen section, a portion of each dissected specimen was immediately embedded in optimal cutting temperature (OCT) compound (Tissue-Tek; Miles Inc., Elkhart, IN) and snap frozen on dry ice and then stored at –80 °C. A panel of mouse monoclonal antibodies to CD4, CD8, CD30, IFN-γ were used (Table 2). Briefly, consecutive 5-μm-thick frozen sections were air dried, fixed in acetone for 20 minutes at room temperature, and rehydrated in phosphate buffered saline (PBS). Nonspecific binding was inhibited by incubating the specimens with 5% normal rabbit serum for 30 minutes at room temperature. The sections were incubated with the optimally diluted primary antibody at room temperature for 2 hours, followed by incubation with a peroxidase-conjugated rabbit anti-mouse IgG antibody (Dako, Glostrup, Denmark) for 45 minutes. The bound antibodies were visualized by the addition of diaminobenzidine tetrahydroxychloride. All steps were followed by 3 washes with PBS. Nuclei were counterstained with hematoxylin for 1 second. Isotype matched control mouse IgG1 for CD4, mouse IgG1 for CD8, mouse IgG2a for CD20, mouse IgG1, κ for IFN-γ, mouse IgG3, k for CD30, and mouse IgG1, k for Vs38c were prepared for each antibody. To confirm that an adequate region was examined for immunohistochemistry, the first and last consecutive sections of each specimen were stained with hematoxylin and eosin.

### 2.3 Transmission electron microscopic examination

Transmission electron microscopic evaluation was conducted according to standard protocols. Eighteen specimens were fixed with 2.5% glutaraldehyde in 0.1 M phosphate buffer (pH 7.4) for 4 hours at 4 °C and washed 3 times with 0.1 M phosphate buffer. The samples were then post fixed in 2% osmium tetroxide, dehydrated in a series of ethanol and propylene oxide, and embedded in epoxy resin. One micrometer sections were stained with methylene blue and the appropriate lacrimal gland and conjunctival portions were thin sectioned on an ultratome (LKB; Gaithersburg, MD) with a diamond knife. Sections in the range of gray to silver were collected on 150-mesh grids, stained with uranyl acetate and lead citrate, and examined under an electron microscope (model 1200 EXII; JEOL, Tokyo, Japan).

## 3 Results

### 3.1 Conventional light microscopic and immunohistochemical analysis

**Figure 1:**
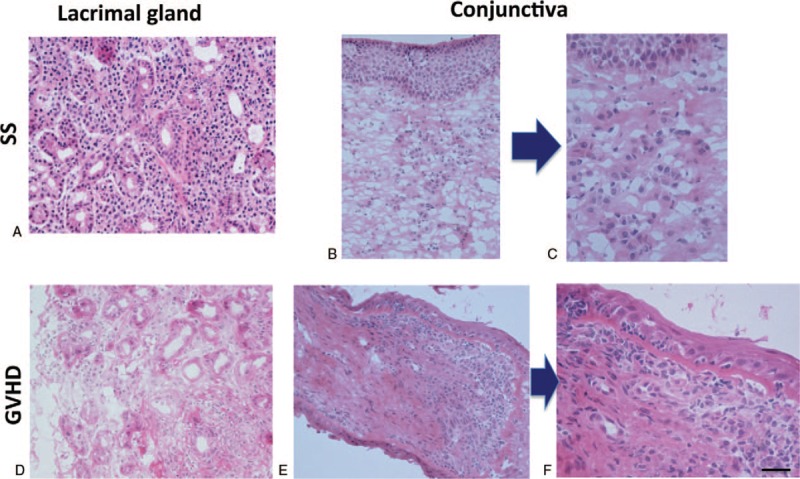
Light microscopic findings of human lacrimal glands and conjunctiva with SS or chronic GVHD. Hematoxylin and Eosin staining. (A) Lacrimal gland affected by SS. Note marked mononuclear inflammatory cell infiltration into the lobules. (B) Conjunctiva disordered by SS. (C) A highly magnified version of (B). (D) Lacrimal gland affected by cGVHD. Note mild inflammatory cell infiltration and excessive fibrosis. (E) Conjunctiva disordered by cGVHD. (F) A highly magnified version of (E). Sample ID: (A, B, C) SS1, (D) GVHD3, (E, F) GVHD2. Scale bar = 100 μm (A, B, D, E), 25 μm (C, F). GVHD = graft-versus-host disease. SS = Sjögren's syndrome.

As indicated by our observations and consistent with our previous report,^[10]^ aberrant accumulation of mononuclear inflammatory cells was associated with the development of SS in the lobules of lacrimal glands (Fig. 1A) and the subepithelial conjunctival stroma (Fig. 1B and C). In contrast, a characteristic feature of myxoedematous fibrosis, which is often seen in the early phase of fibrosis, was observed in the stroma of GVHD-affected lacrimal glands (Fig. 1D) and the subepithelial stroma of GVHD-affected conjunctiva (Fig. 1E and F). There are differences between the 2 diseases of SS and GVHD in the localization of inflammatory cells and the degree of fibrosis.

### 3.2 The distribution and number of B cells and T cells infiltrating lacrimal glands affected by SS

**Figure 2:**
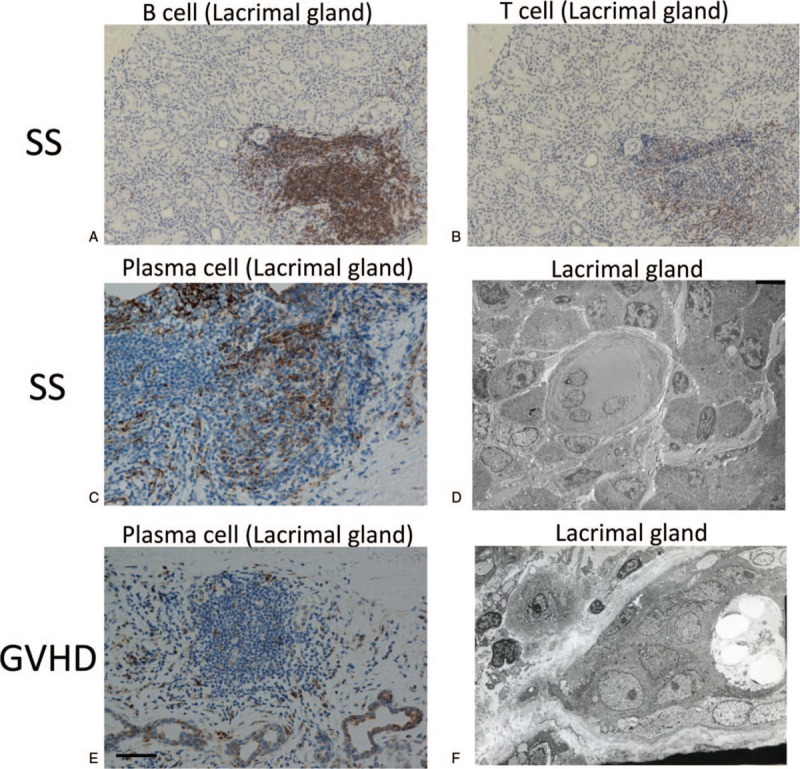
Distribution and degree of infiltrating B cells and T cells on lacrimal glands with SS. (A, B) Lacrimal gland of SS showed more B cell infiltration than T cells. The expression of CD20 (A) and CD45RO (B) is shown in brown. (C, E) Plasma cell infiltrates (Vs38c) in lacrimal gland affected by SS (C) and cGVHD (E) are shown in brown. (E, F) Electronmicrograph of lacrimal gland from a patient with SS (D) and GVHD (F). Note a number of plasma cells in which the nucleus shows cartwheel pattern, a specific feature of plasma cells (D). Sample ID: (A, B, C) SS3, (D) SS1, (F) GVHD3. Scale bar = 100 μm (A, B), 50 μm (C, E) Electron micrograph scale bar = μm (D, F). (D) SS1, (F) GVHD3. Scale bar = 4 μm (D, F). CD = cluster of differentiation, GVHD = graft-versus-host disease, SS = Sjögren's syndrome.

Our experiments indicated that SS-affected lacrimal glands had more B cells (Fig. 2A) than T cells (Fig. 2B). Judging from electron micrographs, plasma cells in SS-affected lacrimal glands (Fig. 2C and D) outnumbered those in their GVHD-affected counterparts (Fig. 2E and F).

### 3.3 Expression of CD4, CD8, and INF-γ on lacrimal glands

**Figure 3:**
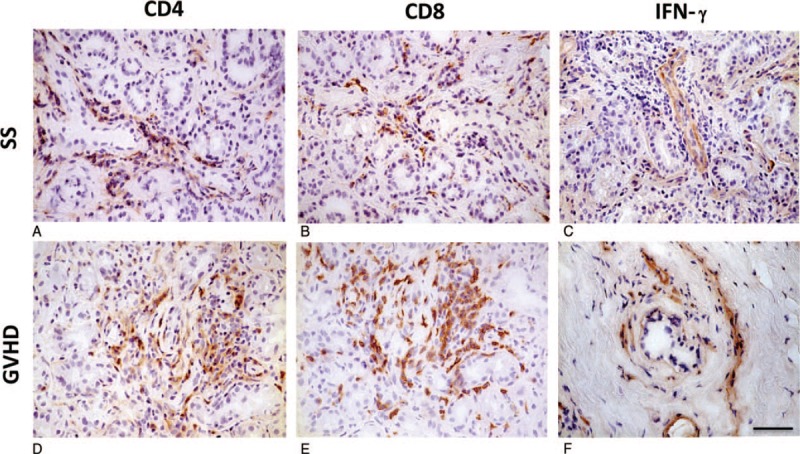
Expression of CD4, CD8, and INF-γ in lacrimal glands. (A, B) CD4 (A) and CD8 (B) expression in lacrimal glands affected by SS. (C) IFN-γ expression on endothelia in lacrimal glands disordered by SS. (D, E) CD4 (D) and CD8 (E) expression in lacrimal gland affected impaired by GVHD. (F) IFN-γ expression on stromal fibroblasts in lacrimal gland affected by cGVHD. Sample ID: (A, B, C) SS2, (D, E) GVHD3, (F) GVHD1. Scale bar = 25 μm (A–F). CD = cluster of differentiation, GVHD = graft-versus-host disease, SS = Sjögren's syndrome.

As reported previously, CD4^+^ T cells (Fig. 3A and D) and CD8^+^ T cells (Fig. 3B and E) were accumulated in both SS- and cGVHD-affected lacrimal glands. IFN-γ was expressed on endothelia of capillaries in lacrimal glands affected by SS (Fig. 3C), whereas it was found on stromal fibroblasts in lacrimal glands disordered by cGVHD (Fig. 3F). These results suggested that IFN-γ expressed on endothelia influenced lymphocyte infiltrates, leading to pathogenic changes.

### 3.4 Expression and localization of CD30 on lacrimal gland in SS

**Figure 4:**
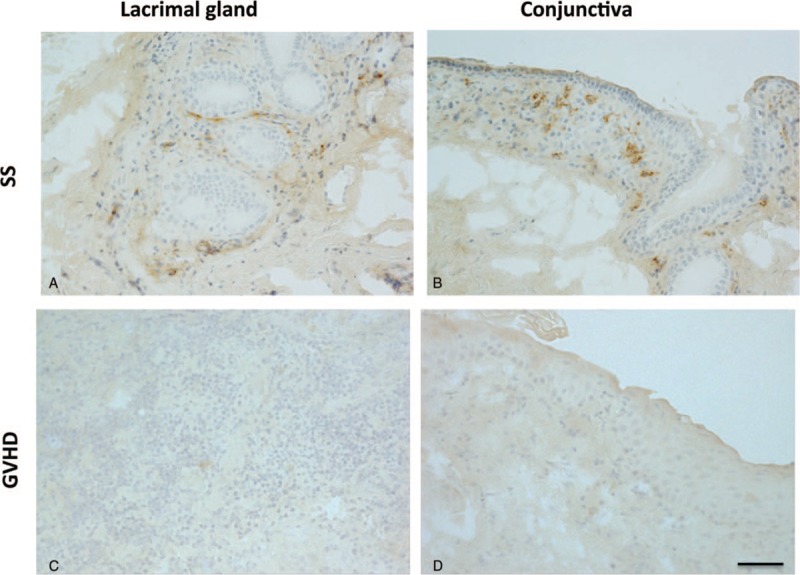
Expression and localization of CD30 in SS- and GVHD-affected lacrimal glands and conjunctiva. (A, B) CD30 expression in lacrimal gland (A) and conjunctiva (B) affected by SS. (C, D) No obvious CD30 expression in lacrimal gland (C) or conjunctiva (D) disordered by GVHD. Sample ID: (A, B) SS1, (C, D) GVHD2. Scale bar = 50 μm. CD = cluster of differentiation, GVHD = graft-versus-host disease, SS = Sjögren's syndrome.

We next examined the localization and expression of CD30, which is presumed to be a prognostic marker of DLBCL. Our observation indicated that CD30^+^ cells infiltrated periductal areas of lacrimal glands affected by SS (Fig. 4A). In addition, CD30^+^ cells were found in subepithelial stroma of lacrimal gland affected by SS and intraepithelial lesions of conjunctiva disordered by SS (Fig. 4B). These phenomena were not clearly observed in GVHD-affected lacrimal glands (Fig. 4C) or conjucntiva (Fig. 4D). As suggested by these findings, CD30^+^ cells existed in ocular surface tissues in SS patients without malignant lymphoma.

## 4 Discussion

A series of our experiments has indicated that CD30^+^ cells, which are diagnostic markers of ML, exist in lacrimal glands and conjunctiva in 3 SS patients without ML. In contrast, this phenomenon was not observed in 3 patients with chronic GVHD-related dry eye disease. In lacrimal glands and conjunctiva in the 3 patients suffering from SS for more than 10 years, there were more B cell and plasma cell infiltrates than T cell infiltrates. IFN-γ was expressed on endothelia in SS-affected lacrimal glands, whereas it was expressed on fibroblastic cells in their cGVHD-affected counterparts. CD30, a 120-kd transmembrane cytokine receptor of the tumor necrosis factor receptor (TNFR) family,^[7]^ is known as a ligand of CD153. It is reported that CD30 is expressed on a subset of activated lymphocytes.^[11]^ In addition, CD30 is a well-known diagnostic marker of classical Hodgkin lymphoma (CHL), anaplastic large cell lymphoma (ALCL), primary mediastinal large B-cell lymphoma and primary effusion lymphoma^[11]^ and a prognostic marker of diffuse large B cell lymphoma.^[7,12]^

In our study, marked mononuclear cell infiltration with CD20^+^ B cells and Vs38c^+^ plasma cells were observed, suggesting that various types of B lymphocyte lineage cells are accumulated excessively in SS lacrimal glands in long-term follow-up patients. It has been shown that autoimmune B cell activation plays a role in the initiation and propagation of SS by producing abnormal antibodies including interferon (IFN)-γ, IL-4, a B cell activating factor and perpetuating chronic inflammation, and also contributes to the development of B cell lymphoma.^[2,13]^ Our experiments indicated that IFN-γ was expressed on endothelia of capillaries in lacrimal glands with SS, whereas the molecule was expressed on interstitial fibroblastic cells in those with GVHD. The expression of IFN-γ is conceivably implicated in innate immunity in SS and contributes to aberrant activation of B cells.^[2]^ In addition, the abnormal activation of B cells is conceived to result in the uncontrolled production of IFN. By using genetic variations outside the MHC locus genes involved in type I interferon pathways, an animal study suggests that B- and T-cell methylation processes are associated with the development of both SS and SS-related lymphoma.^[14]^ Excessive accumulation of B cells and plasma cells are presumed to produce abnormal antibodies in local microenvironments of SS-affected lacrimal glands. Abnormal antibodies may be harmful to lacrimal glands, and thereby lead to the initiation and perpetuation of SS and the development of lymphoma.

With regard to the significance of CD30 expression in lacrimal glands and conjunctiva with SS, lacrimal glands and conjunctiva are representative of exocrine glands (such as pancreas) and mucosal membranes (such as oral mucosa), respectively. CD30 has been recognized as a marker of the malignant Hodgkin and Reed-Sternberg cells in HL.^[15]^ Therefore, CD30^+^ cells on the ocular surface and in lacrimal glands may be an important predictor of malignant lymphoma, and thereby great caution should be accorded to long-term follow-up SS patients.

CD30 is reportedly expressed on T cells, B cells, and eosinophils in association with the development of HL and ALCL.^[5]^ In our investigation into the pathology of SS, B cells are a candidate for expressing CD30. With respect to the malignancy of cells expressing CD30, a determinant of malignant cellular behavior is constitutive expression of CD30 or CD30 ligation. Constitutive CD30 expression is triggered by signal transduction of CD30. Thus, when CD30^+^ cells exist in lacrimal glands and conjunctiva in SS patients without ML, neither constitutive CD30 expression nor CD30 ligation is presumably induced. However, the CD30^+^ cells have the potential to become malignant.^[5]^ Therefore, CD30 expression in SS lacrimal gland and conjunctiva may not be constitutive under nonmalignant condition.

A retrospective cohort study suggested that a focus score of ≥3 in a labial salivary gland biopsy was an independent predictor of the development of non-Hodgkin lymphoma in patients with primary SS.^[16]^ In addition, a germ center-like structure could be referred to as a sign of developing malignant lymphoma in SS patients.^[17]^ It is conceivable that examination of excessive CD30 expression in lacrimal gland and conjunctival tissues may become an additional option to predict the initiation and propagation of ML.

In our study, where the severity of SS was determined based on Green Span classification, Case 2 and Case 3 had grade 3 and 4, respectively. In case 3, CD30-expressing cells with a germ center-like structure were observed. In this case, the germ center like region was closely associated with CD4^+^ and CD8^+^ T cells and CD20^+^ B cells. The international set of classification criteria for primary Sjogren syndrome (SS) from the American College of Rheumatology (ACR) and the European League Against Rheumatism (EULAR)^[18]^ and the revised criteria for the diagnosis of SS proposed by the Japanese Ministry of Health 1999^[3]^ have a subject for lip biopsy for diagnosing SS. It is advisable to detect the CD30 expression on the samples if possible.

There is several study limitations. Firstly, this is a human study with a small number of cases without normal controls due to ethical reason. Therefore, precise mechanistic insights of CD30 expression in SS lacrimal gland and conjunctiva cannot be elucidated. Secondly, we could not evaluate the frequency of CD30 expression in SS general population because of small sample size. Therefore, we should not overestimate our results and interpretation. Further study will be needed to confirm the phenotype of CD30^+^ expressing cells whose shape is round or spindle. In addition, it will be required to show B cell or other cell phenotype which may develop malignant lymphoma. Unfortunately, we could not show those phenotypes due to limitation of our samples in this study.

Further study will be required to elucidate significance of CD30 expression and function in SS patients. Anti CD30 antibody is now available to treat ML. We need to confirm the effectiveness of anti CD30 antibody in SS using SS animal model or elucidate the function^[11]^ and role of CD30 using CD30 knockout mice.

Approximately, 5% of SS patients are reported to develop ML.^[4]^ Earlier work indicated that SS patients were 15 to 20 times more vulnerable to malignant lymphoma (ML) than those without SS.^[2]^ Our novel study suggested that CD30^+^ cells infiltrated lacrimal glands and conjunctiva in SS patients without ML, and therefore reinforced the previous reports. Collectively, clinical settings should accord great caution to the development of ML in patients with SS-related dry eye disease.

## Acknowledgments

The authors thank Dr. Kazuto Yamazaki for critical comments and suggestions, and Drs. Hiroshi Suzuki and Toshihiro Nagai for expert technical assistance.

## Author contributions

**Conceptualization:** Yoko Ogawa, Akiko Ogawa, Masataka Kuwana, Kazuo Tsubota.

**Data curation:** Yoko Ogawa, Akiko Ogawa, Eisuke Shimizu, Masataka Kuwana, Yutaka Kawakami.

**Formal analysis:** Yoko Ogawa, Akiko Ogawa, Shin Mukai, Eisuke Shimizu, Masataka Kuwana, Yutaka Kawakami, Kazuo Tsubota.

**Funding acquisition:** Yoko Ogawa, Shin Mukai, Masataka Kuwana, Yutaka Kawakami, Kazuo Tsubota.

**Investigation:** Yoko Ogawa, Akiko Ogawa, Shin Mukai, Eisuke Shimizu, Masataka Kuwana, Yutaka Kawakami, Kazuo Tsubota.

**Methodology:** Yoko Ogawa, Akiko Ogawa, Shin Mukai, Eisuke Shimizu, Masataka Kuwana, Yutaka Kawakami, Kazuo Tsubota.

**Project administration:** Yoko Ogawa, Akiko Ogawa, Kazuo Tsubota.

**Resources:** Yoko Ogawa, Yutaka Kawakami, Kazuo Tsubota.

**Software:** Yoko Ogawa, Akiko Ogawa, Shin Mukai, Eisuke Shimizu.

**Supervision:** Yoko Ogawa, Masataka Kuwana, Yutaka Kawakami, Kazuo Tsubota.

**Validation:** Yoko Ogawa, Akiko Ogawa, Shin Mukai, Eisuke Shimizu, Masataka Kuwana, Yutaka Kawakami, Kazuo Tsubota.

**Visualization:** Akiko Ogawa, Yoko Ogawa.

**Writing – original draft:** Akiko Ogawa, Yoko Ogawa, Shin Mukai, Masataka Kuwana.

**Writing – review & editing:** Akiko Ogawa, Yoko Ogawa, Eisuke Shimizu, Shin Mukai, Masataka Kuwana, Yutaka Kawakami, Kazuo Tsubota.
